# A systematic review and empiric examination of the conceptual and analytic overlap between allostatic load and multimorbidity

**DOI:** 10.1016/j.ssmph.2026.101962

**Published:** 2026-08-24

**Authors:** Cara L. Booker, Yunlong Liang, Angela Meadows, Owen Cranshaw, Anna Dearman, Evelyn Dilkes, Benedict Hignell, Adisetu Joy Malih, Gabriele Navyte, Meena Kumari

**Affiliations:** a Institute for Social and Economic Research, University of Essex, UK; b Department of Psychology, University of Essex, UK; c Centre for Sustainable Planning and Environments, University of the West of England, UK; d Infection Prevention and Control, Midlands Partnership NHS Foundation Trust, UK

**Keywords:** Allostatic load, Multimorbidity, Systematic review, Comorbidity, Cardiometabolic

## Abstract

**Background:**

Allostatic load is conceptualised as a sub-clinical construct reflecting cumulative physiological wear associated with chronic environmental and psychosocial stress. However, marked heterogeneity in biomarker composition, threshold definition, and medication handling raises concerns about whether different specifications consistently capture a distinct subclinical signal of physiological dysregulation. Unresolved questions remain regarding conceptual and analytic overlap with multimorbidity and related health constructs, particularly when medication use is included in its specification.

**Objective:**

To summarise how population-based studies define and calculate allostatic load, and to assess how these methodological choices affect its relationship with multimorbidity outcomes.

**Methods:**

We conducted a PRISMA-guided systematic review of population-based studies indexed in Web of Science, PsycINFO, MEDLINE, and CINAHL Ultimate in 2022, using the terms “allostasis” and “allostatic load” in post-2010 papers. We then analysed data from four population-based studies to characterise the association between alternative allostatic load specifications and general, cardiometabolic and immune multimorbidity using C-statistic–based measures.

**Results:**

Among 428 studies, we found substantial heterogeneity in operationalisation, particularly regarding biomarker composition, systems, threshold definitions, and medication handling. Empirical analyses showed moderate-to-high overlap between allostatic load and multimorbidity (C-statistics 0.64–0.86), with higher values for cardiometabolic and lower for immune phenotypes and following medication adjustment. Using different threshold definitions for allostatic load produced minimal changes in these associations.

**Conclusions:**

Allostatic load usefully captures multisystem dysregulation, but current operationalisations insufficiently reflect allostatic dynamics and blur subclinical-clinical boundaries. Future research should prioritise longitudinal designs, broader biomarker coverage, and theoretically coherent, transparent approaches to threshold definition and medication adjustment.

## Highlights

• Review findings showed substantial heterogeneity in allostatic load operationalisation.
• Empirical findings showed moderate-to-high overlap between allostatic load and multimorbidity.
• There was higher overlap with the cardiometabolic phenotype.
• Adjusting for medication impacted the overlap between allostatic load and multimorbidity.
• Current allostatic load operationalisation may blur sub-clinical dysregulation.

## 1 Introduction

Allostatic load describes the cumulative ‘wear and tear’ associated with the response to chronic or repeated environmental and psychosocial stressors (McEwen, 1998). In practice, it is measured using multi-system indices that capture alterations of neuroendocrine activity, cardiovascular function, and metabolic processes (Seeman et al., 2001). Researchers have often adopted allostatic load as a biomarker of chronic stress and it has been widely examined in its associations with social factors (Johnson et al., 2017; Ribeiro et al., 2018; Suvarna et al., 2020), psychological health and other health outcomes (D'Amico et al., 2020; Evans et al., 2025; Guidi et al., 2021; Parker et al., 2022) and mortality (Hastings et al., 2022) as a mechanism by which the environment ‘gets under the skin’.

Beyond ongoing debates about the optimal operationalisation of allostatic load, including which biomarkers to select and how to combine them into a single index, additional unresolved issues remain. These include the conceptual and analytic overlaps between allostatic load and ageing (Hastings et al., 2022; Whitley et al., 2024), and with other health constructs, particularly multimorbidity (Dominick et al., 2012), which may intersect with allostatic load both conceptually and analytically. A key concern is that current operationalisations of allostatic load may insufficiently distinguish subclinical physiological dysregulation from established clinical disease processes, thereby increasing overlap with related health constructs. Multimorbidity is commonly defined as more than one co-existing long-term health condition in an individual (Skou et al., 2022). It has increasingly become a focus of research as populations age, with important implications for health-care systems which are usually organised based on single conditions. Conceptually, both allostatic load and multimorbidity may represent manifestations of multi-system physiological processes; however, while allostatic load explicitly emphasises cumulative subclinical physiological dysregulation in its hypothetical underpinnings, multimorbidity captures related downstream health manifestations indirectly through the co-occurrence of diagnosed conditions (Whitty et al., 2020).

At the operational level, allostatic load indices often incorporate biomarkers that have clinical cut-points or are targets of clinical care and medications. While some studies use the clinical cut-points to identify those at high risk, other use sample-based cut-points, however these tend to be at or in some cases above the clinical cut-points. Use of these thresholds or medications has the potential to embed the clinical disease status into the exposure definition itself. A recent systematic review reported that over half of studies included derived allostatic load scores from high-risk quartiles of the sample distribution, while others relied on clinical cutoffs or other approaches (Carbone et al., 2022). However, sample-based cutoffs may vary considerably across populations and may fall close to, within or above established clinical thresholds, thereby blurring the intended distinction between subclinical physiological dysregulation and manifest disease. HDL, waist circumference and waist-to-hip ratio have sex-based cut-points. The formative Lancet 2021 paper identified ethnicity-specific cut-points for BMI (Caleyachetty et al., 2021). Additionally, many biomarkers have been shown to have sociodemographic risk factors such as age, sex, height, ethnicity and socioeconomic position (Robertson et al., 2024). When risk categories or cut-points are sample-based, additional risk factors and sample-specific cut-points need to be considered. Clinical cutoffs, on the other hand, offer greater interpretability but may preferentially capture downstream clinical risk rather than the earlier, subclinical processes central to allostatic load theory (Kezios et al., 2022; Seeman et al., 2001). The Lancet paper highlights how incorporation of sample characteristics may impact cut-points and associated risk (Caleyachetty et al., 2021). As a result, different operationalisation strategies may differentially position allostatic load along a continuum from subclinical dysregulation to overt multimorbidity, with important implications for interpretation. This was shown in Hastings et al. (Hastings et al., 2022), which found a wide variation allostatic load operationalisation, including cut-points and biomarkers used, which were differentially associated with exposures, functioning and mortality risk.

A further methodological challenge arises from the treatment of individuals taking medication in the calculation of allostatic load. Despite the widespread use of antihypertensives, lipid-lowering agents, and other long-term medications in older populations, implications of medication adjustment for the ability of allostatic load to capture subclinical physiological dysregulation have received limited attention in existing systematic review (Carbone et al., 2022; Evans et al., 2025; Lenart-Bugla et al., 2022; Obeng-Gyasi et al., 2023; Parker et al., 2022). A recent consensus statement on the operationalisation of allostatic load highlighted the inconsistent and often inadequate treatment of medication use, particularly for biomarkers such as blood pressure and cholesterol that are regarded as ‘mainstays’ of allostatic load, and underscored the need to account for medication use when constructing allostatic load indices (McCrory et al., 2023). However, incorporating medication use may have limited capacity to differentiate subclinical allostatic processes from treated disease states, potentially obscuring variation in the subclinical allostatic processes that the construct was originally intended to represent.

Here we examined, by systematic review, the construction of allostatic load, focusing on biomarker selection, the extent to which the quantile-based cut-points used fall within published clinical values, and the treatment of medication use. Then, we conducted an empirical analysis of a population-based sample of adults across four datasets to examine how different operationalisations of allostatic load related to general multimorbidity. Allostatic load was operationalised using multiple approaches identified in the literature and the recent consensus statement (McCrory et al., 2023), including both sample-based and clinical cut-points, with and without medication adjustment, as well as a brief five-biomarker score. Finally, we extended our analysis to cardiometabolic and immune multimorbidity. Cardiometabolic multimorbidity was defined as the presence of more than one diagnosed medical condition such as diabetes, hypertension, coronary heart disease, and stroke (Jin et al., 2023). Immune multimorbidity included diagnosed conditions such as asthma, arthritis, emphysema, chronic obstructive pulmonary disease, hyperthyroidism, hypothyroidism, chronic bronchitis and cancer. This focus is warranted because cardiometabolic multimorbidity carries particularly high prognostic significance, being associated with multiplicative increases in mortality risk (Joseph et al., 2022; Zhang et al., 2019). Given that many biomarkers used to construct allostatic load are themselves cardiometabolic, allostatic load may be more closely related to cardiometabolic multimorbidity than to general multimorbidity. Additionally, inflammatory responses have been associated with allostatic load and overall health (Alotiby, 2024; Chen et al., 2018; Yang et al., 2026). While fewer inflammatory biomarkers are collected by studies, it is still important to understand how allostatic load may be related to immune multimorbidity and how any potential overlap compares to either general or cardiometabolic multimorbidity.

## 2 Methods

This systematic review follows the Preferred Reporting Items for Systematic Reviews and Meta-Analyses (Page et al., 2021).

### 2.1 Eligibility criteria

The focus of this review was to examine the composition of allostatic load in population-based cohort studies. Papers were included if they were empirical, included two or more biomarkers, were conducted with human subjects and had sample sizes equal to or greater than 100. Studies were excluded if they were animal studies, case-control, experimental studies or randomised control trials, or non-population-based cohorts (i.e., clinical samples). To reduce potential for bias due to misinterpretation or mistranslation, only articles in English were included.

### 2.2 Data sources and search strategy

A comprehensive literature search was performed by CB in July 2022 in the following electronic databases: Web of Science, PsychINFO, MEDLINE and CINAHL Ultimate. As this paper sought to update the review conducted in 2012 (Beckie, 2012), the search dates were 2010 through June 2022. Only two search terms were used: “allostasis” and “allostatic load.”

### 2.3 Paper selection and data extraction

After deletion of animal studies and any additional duplicates by CB, title and abstract screening was conducted by all authors. Abstracts were split between the review team, with a random 10% of each reviewer's allocation being checked by a different reviewer. Where disagreement occurred (n = 1), the paper was included for full-text inspection. When the eligibility for inclusion of an paper could not be determined based on title and abstract, the paper was included for full-text inspection.

Records (e.g., peer and non-peer-reviewed publications, reports, dissertations, etc.) were divided between the review team. Each record was entered into an online database (Airtable.com). Two separate files were created within the Airtable platform for logistical reasons, and the review team was split between the databases. The following data were extracted for each record: title, authors, year of publication, and DOI or URL; study design, i.e., cross-sectional or longitudinal), recruitment method, population characteristics, and sample size; dataset used (i.e., cohort or author-collected), biomarkers included, method of calculating allostatic load, and where cut-points were used, whether these were clinical or empirical, and their values, if reported, and whether adjustments were made for medication use. All authors were involved in discussions where questions arose. Where a full-text paper could not be obtained, or where additional clarifications were required, attempts were made to contact authors.

Following data extraction and removal of papers that did not meet inclusion criteria, a smaller group (OC, YL, BH and AD) worked to consolidate and clean the two databases. Cleaning involved combining duplicated columns, standardizing biomarker and unit reporting and checking that all cut-off thresholds were reported and classified as either clinical or non-clinical. MK and AM reviewed the consolidated database and worked together to assign physiological systems to the biomarkers used in individual papers. While some papers identified the physiological systems measured by their biomarkers, assignment criteria were not universal and the same biomarker may be in different systems in different papers. Thus, MK and AM reviewed the physiological system assigned to each biomarker and identified the primary or major associated system. An additional classification of systems into primary, secondary or tertiary operationalisation was based on the theory that primary mediators lead to primary effects, which in turn lead to secondary or intermediary outcomes that, and ultimately to tertiary outcomes representing disease (Juster et al., 2010).

### 2.4 Analyses

Descriptive synthesis of the included papers was conducted in accordance with the Centre for Reviews and Dissemination framework (Tacconelli, 2010). Meta-analysis was unfeasible due to the papers’ heterogeneity about training, outcomes, measures, and analytic approach (Tacconelli, 2010).

### 2.5 Empirical analyses

#### 2.5.1 Study design and population

This cross-sectional analysis drew on four population-based studies: the English Longitudinal Study of Ageing (ELSA) (Banks et al., 2025), the Health and Retirement Study (HRS) (University of Michigan, 2025), the Midlife in the United States Study (MIDUS) (Ryff et al., 2021, pp. 2004–2006), and Understanding Society: the UK Household Longitudinal Study (UKHLS) (University of Essex Institute for Social and Economic Research, 2025a). For ELSA, we used the nurse assessment conducted during wave 4, the first wave that included dehydroepiandrosterone sulfate (DHEAS), a neuroendocrine mediator, carried out between July 2008 and August 2009 (Banks et al., 2025). For HRS, we used the enhanced face-to-face protocol implemented in a split-panel design, with one randomly selected half of respondents assessed in 2006 and the remaining half in 2008 (Sonnega et al., 2014). For MIDUS, analytic data came from the MIDUS 2 Biomarker Project collected in 2004–2009 (Ryff et al., 2022). For UKHLS, we used interviews from waves 2–3, with fieldwork spanning 2010–2012, during which nurse visits were carried out for biomarker collection (University of Essex Institute for Social and Economic Research, 2025b). Detailed study designs on these four datasets are summarised in Supplementary data online, Methods. All contributing studies received ethics approval and obtained written informed consent from participants, as documented in their respective user guides and technical reports.

#### 2.5.2 Outcomes

We examined three primary outcomes: general multimorbidity, cardiometabolic and immune multimorbidity. General multimorbidity was defined as the presence of two or more chronic conditions from the list of self-reported diseases available in each dataset (Table S1; Supplementary materials online, Methods) (Chowdhury et al., 2023). Cardiometabolic multimorbidity was defined as the co-occurrence of at least two conditions within the cardiometabolic domain. Specifically, cardiometabolic multimorbidity in MIDUS included heart disease, high blood pressure, circulation problems, blood clots, heart murmur, stroke or transient ischemic attack, anaemia or other blood disease, cholesterol problems, and diabetes. In UKHLS, cardiometabolic multimorbidity comprised congestive heart failure, coronary heart disease, angina, heart attack or myocardial infarction, stroke, diabetes, and high blood pressure or hypertension. For ELSA, cardiometabolic multimorbidity included high blood pressure or hypertension, angina, heart attack, congestive heart failure, heart murmur, abnormal heart rhythm, diabetes or high blood sugar, high cholesterol, and stroke. In HRS, cardiometabolic multimorbidity was defined based on high blood pressure, diabetes, heart problems, and stroke.

Immune multimorbidity was defined as the co-occurrence of at least two conditions within the immune or cancer domains. These included chronic lung disease, asthma, arthritis, cancer or malignant tumour, anaemia or other blood disease, emphysema/Chronic obstructive pulmonary disease, thyroid disease, including hyperthyroidism and hyperthyroidism, and chronic bronchitis. The specific diseases used for each study are indicated in Table S1.

None of the studies included neuroendocrine disorders in their lists of diagnosed medical conditions thus it was not possible to include a neuroendocrine multimorbidity measure in this analysis.

#### 2.5.3 Computation of allostatic load index

Allostatic load was constructed separately within each dataset using biomarkers commonly recommended for its measurement, with scoring procedures harmonised identified from the literature harmonised above across studies. For all versions, biomarkers were coded as high-risk when indicating physiological dysregulation (1 = high-risk, 0 = otherwise), with cut-points defined separately for each biomarker from dataset-specific distributions after excluding respondents with CRP >10 mg/L (to remove acute inflammation) and excluding missing biomarker values. Values were then summed to create participant-level scores. Eight versions of allostatic load were generated (McEwen, 1998): the summation of high-risk biomarkers (Seeman et al., 2001); the same as (McEwen, 1998), but incorporating medication use so that pharmacologically controlled biomarkers were still coded as high-risk (Johnson et al., 2017); a version combining biomarkers with established clinical thresholds and those without (Ribeiro et al., 2018); the same as (Johnson et al., 2017) with medication incorporated (Suvarna et al., 2020); a brief score based on CRP, resting heart rate, HDL-cholesterol, waist-to-height ratio, and HbA1c (McCrory et al., 2023); (D'Amico et al., 2020) the brief score with medication incorporated (Evans et al., 2025); common biomarkers across the four studies with risk quartiles based on the pooled sample and (Guidi et al., 2021) the same as (Evans et al., 2025) with medication incorporated. Detailed information on biomarker selection, risk quantile cut-offs, and the incorporation of medication data in the computation of allostatic load is provided in Table S2 and the supplementary materials online, methods section.

#### 2.5.4 Statistical analysis and covariates

The covariates included age, sex, ethnicity, and survey year. Age was treated as a continuous variable. Sex was classified as male or female. Ethnicity was divided into two categories: White and non-White. Survey year was modelled as a categorical variable to reflect the timing of data collection.

We assessed the extent to which different operationalisation of allostatic load reflects subclinical physiological dysregulation rather than clinically manifest disease using the C-statistic and the paired change in C-statistic (ΔC-statistic). This quantifies the probability that individuals with multimorbidity have higher allostatic load scores than those without. Thus, it provides a measure of empirical overlap: higher values indicate that allostatic load aligns more closely with clinically evident disease rather than earlier, subclinical dysregulation. For descriptive interpretation, C-statistic values were taken to reflect empirical overlap between allostatic load and multimorbidity, with values ≥ 0.9 indicating very high overlap, 0.8-0.89 high, 0.7-0.79 moderate-to-high, 0.6-0.69 moderate, and 0.5-0.59 low overlap (Chowdhury et al., 2025; Schneider et al., 2022). If a given operationalisation of allostatic load incorporated biomarker thresholds or medication information that reflected clinical status, a model will yield a high C-statistic, indicating that the resulting allostatic load index is positioned closer to clinically manifest disease rather than capturing distinct subclinical physiological dysregulation. To further test whether alternative allostatic load operationalisations differ in how they position allostatic load along the subclinical-clinical continuum, we compared each specification with a reference and reported the paired ΔC-statistic: negligible ΔC-statistics indicated similar positioning of allostatic load, whereas positive or negative ΔC-statistics implied a shift toward or away from clinically anchored representations of physiological dysregulation relative to the reference. We conducted these analyses for general, cardiometabolic and immune multimorbidity.

For each dataset and for each outcome, general multimorbidity and cardiometabolic multimorbidity, we fit logistic regression models including one allostatic load specification as the focal predictor and adjusting for age, sex, ethnicity, and survey year. Adjusted predicted values were used to compute the C-statistic with 95% CIs via the DeLong method (DeLong et al., 1988). To evaluate whether alternative allostatic load operationalisations are differentially positioned along the subclinical-clinical continuum relative to a common reference, we prespecified the baseline allostatic load count, defined as the sum of all high-risk biomarkers without medication adjustment, as the reference model, and computed ΔC-statistic for each alternative allostatic load (medication-adjusted, hybrid clinical/empirical, five-item brief, and their medication-adjusted counterparts). We reported the C-statistic for each allostatic load model, the ΔC-statistic and their 95% CIs versus the reference allostatic load. Analyses were conducted in R Studio.

## 3 Results

### 3.1 Search results

**Fig. 1:**
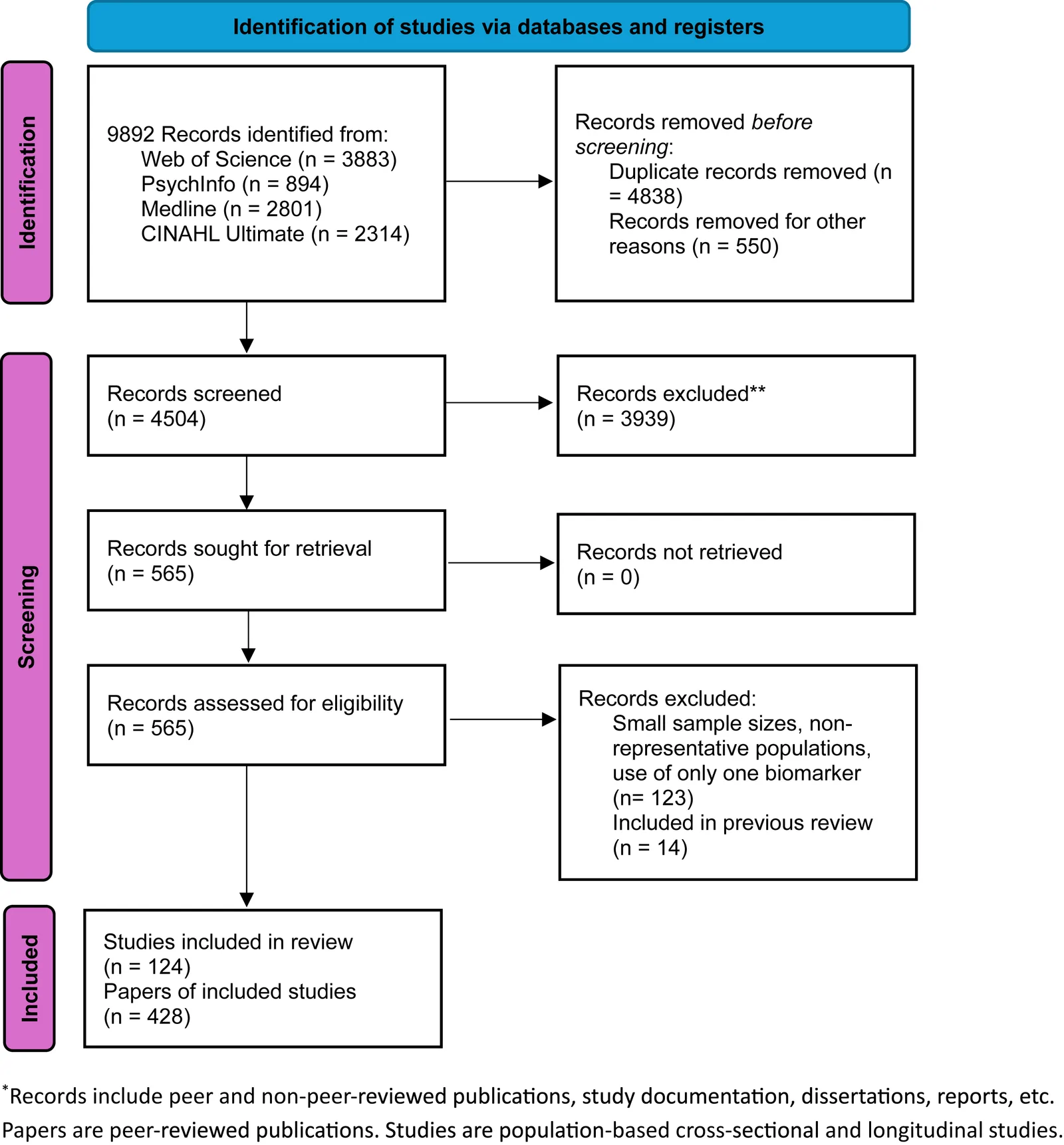
PRISMA diagram* *Records include peer and non-peer-reviewed publications, study documentation, dissertations, reports, etc. Papers are peer-reviewed publications. Studies are population-based cross-sectional and longitudinal studies.

A summary of the systematic search and study selection results is presented in a flow diagram (Fig. 1) in accordance with PRISMA 2020. Across all databases, 9892 records (papers, dissertations, reports, etc.) were identified. After duplicates and animal papers were removed, 4504 titles and abstracts were screened, after which 565 full texts underwent final evaluation for inclusion. 208 papers were excluded for the following reasons: not meeting minimum sample size requirements, studies of clinical populations or included only one biomarker (*n* = 123), or having been included in the previous review (*n* = 14) (Beckie, 2012). Thus, the final sample for analysis comprised 428 papers.

### 3.2 Operationalisation of allostatic load

The number of biomarkers used in the included papers in construction of allostatic load, ranged from two to 28, and the number of physiological systems assessed ranged from one to seven. The most frequently applied biomarker systems were metabolic (n = 414), cardiovascular (n = 413), and inflammation (n = 328). In terms of combinations of systems in allostatic load operationalisation, among the 428 papers, most included both metabolic and cardiovascular systems (n = 410), as shown in Table 1. Only three studies included cardiovascular markers without metabolic markers, four included metabolic markers without cardiovascular markers, and 11 included neither. Table S3 provides the detailed breakdown underlying Table 1. The most common combination, used in 105 papers, includes cardiovascular, HPA-axis/neuroendocrine, immune/inflammation, and metabolic systems. Other dominant combinations spotlight the significant role of cardiovascular systems, often paired with immune/inflammation and metabolic systems, featured in 86 papers. Notably, the complexity of system combinations in allostatic load operationalisation showcases a broad spectrum, with the cardiovascular system being a conspicuous component in most combinations.

**Table 1:** Distribution of studies by inclusion of cardiovascular and metabolic systems.

Category	n
Studies incorporating both cardiovascular and metabolic systems	410
Studies incorporating cardiovascular but not metabolic systems	3
Studies incorporating metabolic but not cardiovascular systems	4
Studies incorporating neither cardiovascular nor metabolic systems	11
**Total**	**428**

Table S4 presents the various biomarker combinations utilised in papers, with each combination being used more than 5 times out of a total of 287 possible combinations. The results underscore a noticeable heterogeneity in the selection of biomarkers across the studies and indicate that there is yet to be a unanimous consensus on a standard set of biomarkers. Notably, certain biomarkers are prominently featured across a majority of the papers. Systolic (n = 396) and diastolic (n = 362) blood pressure were the most used biomarkers, followed by C-reactive protein (n = 317). Table S5 shows that the operationalisation of allostatic load was most often based on a count risk score, which appeared in most studies. Alternative approaches were much less common, including standardised scores, system-level risk scores, or dummy-coded versions of count scores. A smaller number of studies employed more complex strategies such as path or measurement models, system-specific indices, or case-based profiles.

### 3.3 Clinical cut-points and medications

A description of the mean values of all studies with which include the listed biomarkers, those who use non-clinical and the clinical cut-points and the diseases that are diagnosed via clinical cut-points (as identified through the FDA Table of Surrogate Endpoints (U.S. Food and Drug Administration, 2026)) fall are shown in Table 2. Forty-six papers used sex or age-specific cut-points. Sources for cut-points were not often cited, however some papers did sight previous research conducted on the same dataset, study user guides and consensus statements issued by various societies and associations. Quantile thresholds for diastolic blood pressure (83 mmHg) and fasting glucose (7.4 mmol/L) exceeded their corresponding clinical cut-points (80 mmHg and 7 mmol/L, respectively). For body mass index, the quantile cutoff (28.5 kg/m^2^) was above the overweight threshold (25 kg/m^2^). Those for HbA1c (5.8% vs. 6.5%), LDL-cholesterol (3.4 vs. 4.9 mmol/L), and total cholesterol (5.5 vs. 7 mmol/L) fell below clinical criteria. Systolic blood pressure (137 mmHg) and HDL-cholesterol (1.22 mmol/L vs. <1 mmol/L) showed values close to clinical thresholds.

**Table 2:** Top cut-points used in the allostatic load algorithms.

Biomarkers	Studies (n)	Mean values		Clinical cut-points	Diagnosed Disease(s)
		All	Did not use clinical cut-points		
Systolic blood pressure	231	136.9	137 (n = 115)	140	Hypertension, Hypotension, Inoperative haemorrhage
Diastolic blood pressure	222	84.3	83 (n = 108)	80	
HbA1c	174	6.5	5.8 (n = 94)	6.5	Type 1 diabetes mellitus, Type 2 diabetes mellitus, Lipodystrophy
HDL-cholesterol	187	1.17	1.22 (n = 90)	< 1	N/A
LDL-cholesterol	76	3.45	3.37 (n = 45)	4.9	Hypercholesterolemia, Lysosomal Acid Lipase (LAL) deficiency
Total cholesterol	127	7.71	5.5 (n = 54)	7	N/A
Fasting glucose	92	6.94	7.42 (n = 54)	7	Lipodystrophy
Body Mass Index	156	28	28.5 (n = 77)	25	N/A

**Fig. 2:**
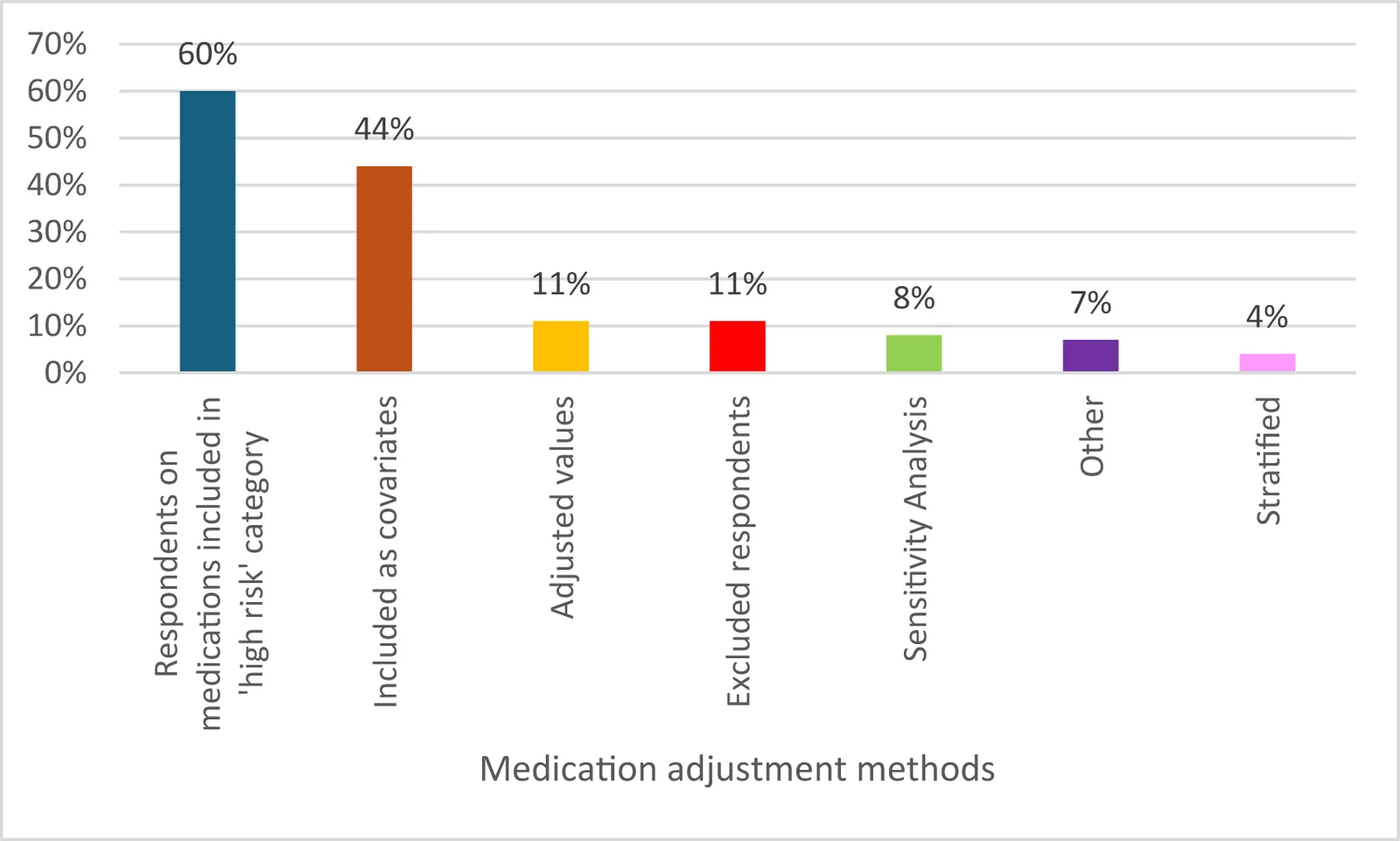
Distribution of medication adjustment methods across paper.

Fig. 2 illustrates the different approaches used to adjust for medications in the construction of allostatic load. The majority of papers (∼60% addressing medications) assigned participants who used relevant medications into the highest risk category for that biomarker, although some assigned these participants to the second highest risk category for certain biomarkers. Controlling for use of medication was the second most common method (∼44%). Amongst the papers that included sensitivity analysis three reclassified those on medication as high risk for the biomarkers the medication may have an impact on, three excluded those on medication and two controlled for use of medication. The seven papers that used other methods either excluded those on other medication but not medication that may have a specific impact on the biomarkers of interest, i.e. specific psychiatric medications, indirectly controlling for medications or experimented with different cut-point values.

### 3.4 Empirical analysis of the overlap between allostatic load and multimorbidity

As shown in Table 3, 17,231 adults were analysed: 2482 from ELSA (mean age 62.7 years, 52.6% female), 8431 from HRS (mean age 67.7 years, 58.9% female), 908 from MIDUS (mean age 57.7 years, 56.7% female), and 5410 from UKHLS (mean age 52.4 years, 55.5% female), after excluding participants with CRP >10 mg/L or with missing data on sociodemographic factors, multimorbidity and medication, or allostatic load. Overall, the mean age was 61.6 years (SD 14.0), 56.8% were women, and 88.2% were White. While gender distributions were fairly similar across the studies, the ‘ageing’ studies, ELSA and HRS, were older than the general populations studies, MIDUS and UKHLS. The US-based studies, MIDUS and HRS, were also much more ethnically diverse than the UK-based Studies, ELSA and UKHLS.

**Table 3:** Characteristics of participants by datasets.

	All	ELSA	HRS	MIDUS	UKHLS
	*N = 17231*	*N = 2482*	*N = 8431*	*N = 908*	*N = 5410*
**Age**	61.6 (14.0)	62.7 (7.34)	67.7 (10.4)	57.7 (11.2)	52.4 (16.2)
**Sex**					
Male	7442 (43.2%)	1176 (47.4%)	3468 (41.1%)	393 (43.3%)	2405 (44.5%)
Female	9789 (56.8%)	1306 (52.6%)	4963 (58.9%)	515 (56.7%)	3005 (55.5%)
**Ethnicity**					
White	15198 (88.2%)	2440 (98.3%)	7047 (83.6%)	739 (81.4%)	4972 (91.9%)
Non-White	2033 (11.8%)	42 (1.69%)	1384 (16.4%)	169 (18.6%)	438 (8.10%)
**Year**					
2004	59 (0.34%)			59 (6.50%)	
2005	217 (1.26%)			217 (23.9%)	
2006	4148 (24.1%)		3949 (46.8%)	199 (21.9%)	
2007	241 (1.40%)			241 (26.5%)	
2008	5814 (33.7%)	1180 (47.5%)	4482 (53.2%)	152 (16.7%)	
2009	1342 (7.79%)	1302 (52.5%)		40 (4.41%)	
2010	1573 (9.13%)				1573 (29.1%)
2011	2669 (15.5%)				2669 (49.3%)
2012	1168 (6.78%)				1168 (21.6%)
**Allostatic load (reference)**	3.21 (2.49)	3.26 (2.28)	2.49 (1.79)	6.68 (3.94)	3.71 (2.58)
**Allostatic load (med-adjusted)**	4.23 (2.87)	4.01 (2.78)	3.96 (2.35)	8.33 (4.60)	4.07 (2.77)
**Allostatic load (five-item)**	1.23 (1.14)	1.13 (1.10)	1.24 (1.11)	1.30 (1.20)	1.26 (1.21)
**Allostatic load (five-item, med-adjusted)**	1.71 (1.30)	1.41 (1.24)	1.96 (1.28)	1.77 (1.34)	1.45 (1.27)
**Allostatic load (clinical cutoffs)**	3.65 (2.53)	3.52 (2.37)	3.13 (1.89)	7.20 (4.02)	3.92 (2.65)
**Allostatic load (clinical cutoffs, med-adjusted)**	4.62 (2.93)	4.24 (2.85)	4.58 (2.46)	8.45 (4.59)	4.23 (2.84)
**General multimorbidity (binary)**					
No	9452 (54.9%)	1599 (64.4%)	3215 (38.1%)	144 (15.9%)	4494 (83.1%)
Yes	7779 (45.1%)	883 (35.6%)	5216 (61.9%)	764 (84.1%)	916 (16.9%)
**Cardiometabolic multimorbidity (binary)**					
No	13739 (79.7%)	2027 (81.7%)	5967 (70.8%)	629 (69.3%)	5116 (94.6%)
Yes	3492 (20.3%)	455 (18.3%)	2464 (29.2%)	279 (30.7%)	294 (5.43%)
**Immune multimorbidity (binary)**					
**No**	15357 (89.1%)	2352 (94.8%)	7075 (83.9%)	787 (86.7%)	5143 (95.1%)
**Yes**	1874 (10.9%)	130 (5.2%)	1356 (16.1%)	121 (13.3%)	267 (4.9%)

Note: values represent either mean with standard deviation in brackets or total number of participants with column specific percentages in brackets.

**Fig. 3:**
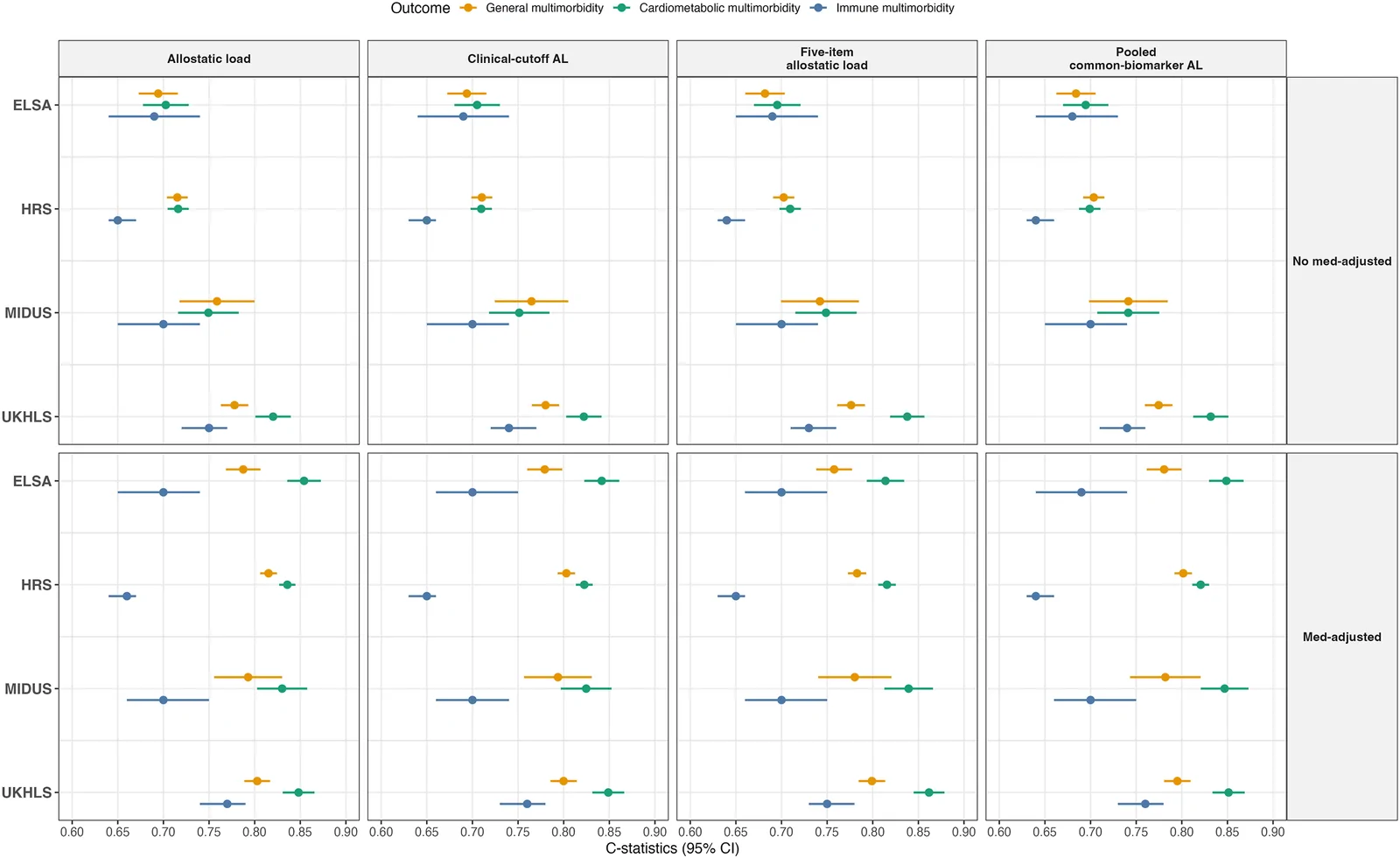
C-statistics for alternative specifications of allostatic load in relation to general and cardiometabolic multimorbidity across cohorts.

Mean levels of allostatic load varied across operationalisations and datasets. Consistent with the predominant operationalisation identified in the systematic review, the reference specification applied a high-risk quartile approach without medication adjustment. The overall mean was 3.21 (SD 2.49), with dataset means of 3.26 in ELSA, 2.49 in HRS, 6.68 in MIDUS, and 3.71 in UKHLS. With medication adjustment, the overall mean increased to 4.23 (SD 2.87), with dataset means of 4.01, 3.96, 8.33, and 4.07, respectively. For the clinical cutoff version, the overall mean was 3.65 (SD 2.53) without medication adjustment and 4.62 (SD 2.93) with medication adjustment, with dataset means ranging from 3.13 to 7.20 and 4.24 to 8.45, respectively. For the five-item index, the overall mean was 1.23 (SD 1.14) without medication adjustment and 1.71 (SD 1.30) with medication adjustment, with dataset means ranging from 1.13 to 1.30 and 1.41 to 1.96, respectively. General multimorbidity was present in 45.1% of participants overall, ranging from 16.9% in UKHLS to 84.1% in MIDUS. Cardiometabolic multimorbidity, observed in 20.3% overall, ranged from 5.4% in UKHLS to 30.7% in MIDUS. Immune multimorbidity was observed in 10.9% of all participants and proportions ranged across studies from 4.9% in UKHLS to 16.1% in HRS. As shown in Fig. 3 and Supplementary Table S6, the cross-sectional C-statistics for the association between allostatic load and multimorbidity ranged from moderate to high (approximately 0.64-0.86). C-statistics ranged from 0.68 to 0.82 for general multimorbidity, from 0.69 to 0.86 for cardiometabolic multimorbidity, and from 0.64 to 0.77 for immune multimorbidity. Also, higher C-statistics were generally observed for cardiometabolic multimorbidity than for general multimorbidity across most allostatic load specifications (ΔC = −0.01 to +0.07), indicating greater empirical overlap. Conversely, immune multimorbidity C-statistics were generally lower than either general or cardiometabolic multimorbidity. This suggests less overlap between allostatic load specifications and immune multimorbidity. When medications were not included in the allostatic load calculation, the C-statistics for both general and cardiometabolic multimorbidity tended to be higher in UKHLS and MIDUS compared with ELSA and HRS, whereas these between-cohort differences were substantially attenuated after medication adjustment.

Medication adjustment was associated with higher C-statistics across all four studies, although the magnitude of improvement varied by cohort (Fig. 3; Supplementary Table S6). Moreover, the increase in C-statistics was larger for cardiometabolic multimorbidity than for either general or immune multimorbidity. The largest increase was observed in ELSA and HRS, where ΔC ranged from +0.06 to +0.15 across outcomes and specifications. In contrast, MIDUS and UKHLS showed more modest increments, with ΔC ranging from +0.02 to +0.09.

C-statistics based on clinically defined cut-points, the five-item and the pooled common-biomarker allostatic load indices were comparable to those based on distribution-based allostatic load specifications. The clinical cutoff allostatic load without medication adjustment offered little divergence from the reference allostatic load (ΔC ≈ 0 in all datasets, Supplementary Table S6). When medication adjustment was added, its C-statistics closely tracked those of the medication-adjusted allostatic load. C-statistics of the five-item and pooled common-biomarker allostatic load indices were nearly identical to the reference specification of allostatic load, and when medication was incorporated, the results closely paralleled those of the medication-adjusted allostatic load.

## 4 Discussion

This review of 428 studies examines how allostatic load has been operationalised, with particular attention to the use of sample-specific versus clinical thresholds and the handling of medication use. In terms of biomarker systems, cardiovascular and metabolic markers were included in the vast majority of studies, whereas fewer than half incorporated primary mediators of allostatic load, such as neuroendocrine biomarkers, consistent with prior review (Johnson et al., 2017). Often the selection of biomarkers used to calculate allostatic load is limited to what an individual study has collected. With respect to thresholds, when sample-based cut-points were used, those for diastolic blood pressure, fasting glucose, and body mass index were typically set higher than clinical cut-points, while thresholds for other biomarkers with established clinical values (such as HbA1c, LDL cholesterol, and total cholesterol) were often lower. Regarding medication use, about a third of studies reported how it was addressed. Most classified participants on relevant medications as high risk for the corresponding biomarker, while others used alternative strategies, including assigning them to the second-highest risk group, controlling for medication use, conducting sensitivity analyses, excluding certain medication groups, or experimenting with different cut-off values.

Our empirical analyses indicated that C-statistics based on clinically defined thresholds, a pooled common-biomarker and a simplified allostatic load index were comparable to those obtained using sample-based allostatic load measures, consistent with previous literature showing relatively small differences in predictive performance for age-related health outcomes across allostatic load scoring algorithms (McLoughlin et al., 2020). Nevertheless, results should be interpreted with caution in relation to multimorbidity. Although sample-based quantile methods are widely used to define high-risk ranges for allostatic load, quantile-based thresholds may fall close to or within established clinical cut-points (Bruun-Rasmussen et al., 2022; Robertson et al., 2017), which may shift the measure toward clinical risk indicators rather than maintaining a clear subclinical dimension, thereby influencing interpretation of allostatic load (Carbone et al., 2022). Our review showed that in certain biomarkers, population-specific cut-points exceeded established clinical thresholds, whereas in others with well-defined clinical cut-offs, quantile-based cut-offs remained within the clinically normal range. In the context of multimorbidity, quantile-based approaches may encompass both clinical risk and subclinical dysregulation. Therefore, when different allostatic load algorithms demonstrate comparable results, interpretation should take into account the underlying threshold definitions and biomarker composition, with careful consideration of whether clinical disease information may be indirectly incorporated. Additionally, the characteristics of the sample that may impact clinical cut-offs should also be considered. Several biomarkers have gender-based cut-points and ethnicity-based cut-points have been suggested for BMI (Caleyachetty et al., 2021; Robertson et al., 2024). Studies should also consider other sociodemographic characteristics that have been associated with biomarker levels.

Furthermore, our results indicated that the current allostatic load indices showed moderate-to-large informational overlap with multimorbidity. On the one hand, this result may partly reflect measurement-level overlap, in that the operationalisation of allostatic load captured the physiological embodiment of existing chronic conditions, which may be reflected in altered biomarker profiles in cross-sectional settings. On the other hand, it may also point to shared underlying pathophysiological processes involving cross-system physiological dysregulation. Mechanistically, allostatic load may represent an evolving response to chronic stress, perhaps leading to evolving set points that provide a link between stressful exposures, ageing and multimorbidity (Ramsay & Woods, 2014; Skou et al., 2022). It can be speculated that multimorbidity represents the end point or clinical expression of allostatic load (Mira et al., 2025). As such, this result did not negate the conceptual validity of allostatic load as a measure of multisystem physiological dysregulation but instead underscored the limitations of its current operationalisation in adequately capturing the dynamic nature of physiological regulation. To understand this more fully, it is necessary to measure markers of allostatic load over time to better understand allostasis, allostatic load and multimorbidity.

In most studies and model specifications, allostatic load showed higher C-statistics for cardiometabolic multimorbidity than for general multimorbidity. This pattern is consistent with prior studies linking allostatic load to cardiometabolic multimorbidity (Hong et al., 2026; Liu et al., 2025), while further suggesting systematic differences in the degree of informational overlap between allostatic load and different multimorbidity phenotypes. From a clinical phenotype perspective, cardiometabolic multimorbidity are typically characterised by well-defined, measurable, and highly standardised biomarker abnormalities, many of which constitute core components of current operationalisations of allostatic load (Juster et al., 2010; McEwen, 2004). From a mechanistic perspective, cardiometabolic functioning represents a downstream domain of stress-related physiological dysregulation, in which multisystem alterations may be more readily detectable (Juster et al., 2010). Conversely, our findings found lesser overlap between allostatic load and immune multimorbidity which could reflect the selection of or the relative lack of cut-points for many of the inflammatory biomarkers collected by the study These findings highlight the importance of distinguishing between the pathophysiological processes indexed by allostatic load and their expression in specific clinical phenotypes when interpreting associations with multimorbidity.

Additionally, the brief five-item allostatic load index showed comparable results to more extensive allostatic load operationalisations in relation to multimorbidity, suggesting a high degree of overlap in the information captured, even when additional biomarkers such as systolic and diastolic blood pressure and total cholesterol were included. This is consistent with multi-cohort evidence that broader biomarker batteries often add limited incremental predictive information beyond a small core of metabolic-inflammatory markers, while greater standardisation in biomarker selection may improve cross-cohort comparability (McCrory et al., 2023). The convergence may reflect a broader characteristic of current allostatic load operationalisations, which tend to be driven by downstream metabolic and inflammatory alterations. As a result, both brief or expanded allostatic load indices may overlap with subclinical or clinical disease processes, thereby insufficiently representing the dynamic, multisystem regulatory burden associated with chronic stress. These findings underscore the need to consider where specific allostatic load operationalisations are situated along the continuum from early physiological dysregulation to manifest disease, and whether alternative approaches are required to better capture subclinical allostatic dynamics. Collection of biomarkers across the life course could also shed light on the quality of life and the severity of multimorbidity onset as well as the more immediate impacts of life stressors and role changes over with time. The theoretical underpinning of allostatic load as a measure of chronic stress could suggest impacts on disease severity and functioning. A recent paper by Wen et al. found associations between measures of biological ageing as measured by biomarkers and functioning while also advocating for collection of biomarkers across the life course (Wang et al., 2026).

We further examined the impact of incorporating medication use into the operationalisation of allostatic load. Results showed the inclusion of medication information was associated with increased overlap between allostatic load and multimorbidity. Medication use can modify the levels of biomarkers that constitute allostatic load (Booth et al., 2013), yet there is no consensus on how medications should be treated in the operationalisation of allostatic load. We found that the majority of studies did not report how medications were treated in the construction of allostatic load, as only 36% of papers included a report of medications. The difficulties of addressing medication in these studies was discussed recently in a consensus definition of allostatic load, which cautioned that the ‘mainstays’ in allostatic load biomarkers such as blood pressure and cholesterol, included in over 90% of studies reviewed here, may not be ideal components in studies of older adults unless medication use is explicitly considered (McCrory et al., 2023). Our empirical findings extend this methodological discussion from a complementary perspective. Specifically, incorporating medication information may correct for treatment-related masking of biomarker values but substantially increases the overlap between allostatic load and multimorbidity, highlighting a non-negligible methodological trade-off. Such methodological tension becomes particularly salient in settings where cohorts differ markedly in age structure and underlying disease burden, as reflected in the heterogeneous profiles of the populations examined in this study. Consequently, the interpretation of medication-adjusted allostatic load requires careful consideration of study design and cohort-specific characteristics.

Previous studies have rarely operationalised the subclinical features of allostatic load with sufficient care. In mechanism-oriented research, excluding individuals with multimorbidity at baseline is sometimes adopted to reduce construct overlap (Mira et al., 2025). However, recalibrating percentile thresholds within restricted samples effectively redefines the physiological scale associated with high risk, thereby diluting the interpretive clarity of allostatic load as a subclinical mechanistic marker. The handling of medication use further compounds this ambiguity. Additionally, incorporating medication data may correct for treatment-induced normalisation of biomarkers in specific cases, yet it simultaneously introduces information linked to diagnoses, shifting allostatic load towards a more overtly clinical construct. Conversely, ignoring medication use risks systematic underestimation of underlying physiological burden. Consequently, decisions regarding medication adjustment should be explicitly aligned with whether the analytical objective prioritises disease state identification or the characterisation of latent physiological dysregulation.

Despite the strength of combining a systematic review with multi-cohort analyses, our study has several limitations. First, the cross-sectional design precludes establishing temporal ordering, so we cannot establish the extent to which this degree of construct overlap would inflate associations in longitudinal designs. Second, our analyses relied primarily on C-statistics/ΔC to characterise how different operationalisations of allostatic load are positioned along the subclinical-clinical continuum; yet C-statistics alone do not capture the full extent of construct alignment. Third, the datasets span different periods (2004–2012) and populations, with varying disease burdens, treatment environments, and medication patterns, and the samples are predominantly UK/US and White, limiting generalisability. The differences in ethnic diversity between the US and UK-based studies may have had impacts on cut-points. Selection of biomarkers was limited to those collected by these studies and thus more biomarkers could not be included in the pooled common-biomarker specification. Finally, missing data on biomarkers and covariates were handled using complete-case analyses. Although the proportion of missingness was non-trivial, we assumed data were missing at random, conditional on observed covariates, such that the analysed sample can be considered representative of the target population under this assumption. Multiple imputation was not undertaken, given our primary focus on construct overlap rather than parameter estimation.

Overall, our findings suggest that allostatic load remains a useful construct for capturing multisystem physiological dysregulation. However, prevailing approaches to its operationalisation show clear limitations in adequately reflecting the dynamic nature of allostasis and in distinguishing subclinical dysregulation from established clinical disease. Future research should place greater emphasis on longitudinal designs, incorporate biomarkers spanning key regulatory pathways, and adoption of theoretically coherent and transparently reported strategies for threshold definition and medication adjustment. Such advances are essential for more accurately delineating the role of allostatic load in the interrelated processes of stress exposure, ageing, and the development of multimorbidity.

## CRediT authorship contribution statement

**Cara L. Booker:** Writing – review & editing, Writing – original draft, Visualization, Validation, Methodology, Data curation, Conceptualization. **Yunlong Liang:** Writing – review & editing, Writing – original draft, Visualization, Validation, Methodology, Formal analysis, Data curation, Conceptualization. **Angela Meadows:** Writing – review & editing, Writing – original draft, Validation, Methodology, Data curation, Conceptualization. **Owen Cranshaw:** Writing – review & editing, Validation, Data curation. **Anna Dearman:** Writing – review & editing, Validation, Data curation. **Evelyn Dilkes:** Writing – review & editing, Visualization, Data curation. **Benedict Hignell:** Writing – review & editing, Validation, Data curation. **Adisetu Joy Malih:** Writing – review & editing, Validation, Data curation. **Gabriele Navyte:** Writing – review & editing, Validation, Data curation. **Meena Kumari:** Writing – review & editing, Writing – original draft, Validation, Methodology, Data curation, Conceptualization.

## Statement of ethics

The systematic review was based exclusively on published literature and thus ethical consent was not required. The data analysed was from publicly available datasets. These data have been reviewed and informed consent was obtained for all participants. ELSA Wave 4 received ethical approval from the National Hospital for Neurology and Neurosurgery & Institute of Neurology Joint Research Ethics Committee on 12th October 2007 (07/H0716/48). HRS was reviewed by the University of Michigan Health Sciences/Behavioral Sciences Institutional Review Board Protocol: HUM00061128 Approved through 10/18/2018 with associated protocols: HUM00056464, HUM00002562, HUM00074501, HUM00079949, HUM00080925, HUM00085942, HUM00099822, HUM00103072, HUM00106904, HUM00122335, REP00000046. MIDUS Biomarker Project was approved by the University of Wisconsin-Madison Institutional Review Board, Protocol number 2014-0813. The University of Essex Ethics Committee has approved all data collection on Understanding Society main study and innovation panel waves, including asking consent for all data linkages except to health records. Approval for the collection of biosocial data by trained nurses in Waves 2 and 3 of the main survey was obtained from the National Research Ethics Service (Understanding Society – UK Household Longitudinal Study: A Biosocial Component, Oxfordshire A REC, Reference: 10/H0604/2).

## Declaration of competing interests

The authors declare that there are no competing interests in the writing of this manuscript.

## Data Availability

All data from the systematic review and the data analyses are publicly available. Data generated from this study are included in the article or its supplemental materials. Any additional enquiries should be directed to the corresponding author.
