# Supplementary material for: A systematic review and empiric examination of the conceptual and analytic overlap between allostatic load and multimorbidity
Source: SSM Popul Health. 2026 Aug 24;35:101962. doi: 10.1016/j.ssmph.2026.101962 (PMC13572744; doi:10.1016/j.ssmph.2026.101962)
Supplement: Supplementary file 1 [file mmc1.docx]

# Methods

## Study designs of the ELSA, HRS, MIDUS, and UKHLS

The UK Household Longitudinal Study (UKHLS; Understanding Society) is a nationally representative panel survey conducted in the UK.^1^ The first wave began in 2009, recruiting households through a stratified clustered sampling design. Data collection has been carried out annually, and nurse visits were introduced to collect biomarkers and anthropometric measures in wave 2 and wave 3. In the present analysis, we used data from nurse assessments in wave 2 (2010–2011) and wave 3 (2011–2012), during which 13,286 participants attended. After excluding those with C-reactive protein (CRP) levels >10 mg/L (n=706), 12,580 remained. Participants with missing sociodemographic data (n=19), multimorbidity and medication information (n=3722), or incomplete allostatic load biomarkers (n=3429) were sequentially excluded, yielding an analytic sample of 5410 participants.

The English Longitudinal Study of Ageing (ELSA) is an ongoing cohort survey of adults aged 50 years and older in England.^2^ The baseline survey was launched in 2002–2003, with follow-up every two years and nurse visits every four years. Nurse assessments included anthropometry, physical measures, and the collection of blood samples. For this study, data from the wave 4 nurse visit (2008–2009) were used. A total of 8643 individuals attended, of whom 444 were excluded for CRP >10 mg/L. Further exclusions included missing sociodemographic data (n=3), multimorbidity and medication information (n=1817), and allostatic load biomarkers (n=3897), leaving 2482 participants in the final analytic sample.

The Health and Retirement Study (HRS) is a longitudinal study of adults aged 50 years and older in the USA, initiated in 1992.^3^ It employs a nationally representative sampling frame with biennial follow-up surveys. Since 2006, half of respondents have been randomly assigned to enhanced face-to-face interviews (EFTF) that include physical measurements and venous blood collection, with the other half assessed in 2008. For the present analysis, 13,064 participants in 2006 and 2008 contributed biomarker data. We excluded individuals with CRP >10 mg/L (n=1915), followed by exclusions for missing sociodemographic (n=2), multimorbidity and medication (n=123), and allostatic load (n=2593) information, resulting in 8431 participants for analysis.

The Midlife in the United States Study (MIDUS) is a national study of adults examining health and psychosocial factors across the life course.^4^ The MIDUS 2 biomarker project (2004–2009) collected biological and physical measures from respondents at clinical research centres. In total, 1255 individuals participated in biomarker collection. After excluding those with CRP >10 mg/L (n=74), 1181 remained. Additional exclusions were made for missing sociodemographic information (n=3), multimorbidity and medication (n=162), and allostatic load indicators (n=108), leaving 908 participants for analysis.

## Disease ascertainment

**ELSA Wave 4**

Chronic conditions were identified from self-reports of physician diagnoses, using a structured set of rules to determine whether respondents were considered as currently having a condition. Participants were classified as currently having a condition if they reported a new doctor diagnosis in the current wave, if they had reported it previously and confirmed that they still had it, or if they had earlier denied it but now indicated they had the condition. For conditions that included a follow-up question on whether the disease was still present, logically inconsistent answers (for example, no prior report but simultaneously confirming and saying they still had the condition) were coded as missing. Participants were classified as not currently having the condition if it had never been reported, if they had reported it previously but now stated it had resolved, or if they continued to deny it with a reason other than “now have it.”

The following conditions were included: chronic lung disease, asthma, arthritis, osteoporosis, cancer, Parkinson’s disease, psychiatric problems, Alzheimer’s disease, dementia, high blood pressure, angina, heart attack, heart failure, heart murmur, abnormal heart rhythm, diabetes, stroke, and high cholesterol. Conditions such as arthritis, heart attack, diabetes, and stroke did not include the “still have it” follow-up; for these, current status was based on new or confirmed prior reports, with inconsistencies coded as not having the condition.

**UKHLS Waves 2-3**

In waves 2-3, UKHLS covers asthma, arthritis, congestive heart failure, coronary heart disease, angina, heart attack (myocardial infarction), stroke, emphysema, hyperthyroidism/overactive thyroid, hypothyroidism/underactive thyroid, chronic bronchitis, liver disease, cancer or malignancy, diabetes, epilepsy, high blood pressure/hypertension, and clinical depression. UKHLS determines current disease status from respondents’ doctor-diagnosis reports, using parallel items asked at each wave. In wave 1, respondents were asked whether they had each condition and, if yes, whether they still had it. A condition was coded present when it was reported and the respondent said they still had it; it was coded absent when it was reported but no longer present, or not reported at all.

In waves 2 and 3, respondents were asked whether they had received any new diagnosis since the previous interview and, if yes, whether they still had it. A condition was coded present if a new diagnosis was reported and marked as still present, or if it had already been classified as present in a prior wave. A condition was coded absent if a new diagnosis was reported but marked “not still present,” if no new diagnosis was reported and the prior wave did not indicate the condition, or if the “not still present” response was given. For the new entrants, a condition was coded present when it was reported and the respondent said they still had it; it was coded absent when it was reported but no longer present, or not reported at all. For myocardial infarction and stroke in waves 2–3, where the ‘still’ questions were not asked, current-wave reports were coded based on previous reports unless a new diagnosis was reported.

**MIDUS 2 Biomarker Project and HRS 2006/2008**

In the MIDUS study, chronic conditions were assessed directly through the self-administered questionnaire. Participants were asked, “Have you ever had any of the following conditions/illnesses?” followed by a list that included cardiovascular diseases (heart disease, high blood pressure, circulation problems, blood clots, heart murmur, transient ischemic attack or stroke, anaemia, and cholesterol problems), metabolic and respiratory disorders (diabetes, asthma, emphysema/COPD, and thyroid disease), cancers, musculoskeletal conditions (arthritis), gastrointestinal and liver diseases (peptic ulcer disease, cirrhosis/liver disease, colon polyp), neurological and psychiatric conditions (depression, alcoholism, glaucoma), and infections (tuberculosis, positive TB skin test, history of blood transfusion before 1993). Ascertainment was based on self-report of ever having been diagnosed with each condition.

In the HRS, chronic condition data were obtained from the RAND HRS longitudinal file, which harmonises and cleans the original survey reports.^5^ Respondents were asked in this wave whether they had specific conditions, including high blood pressure, diabetes, cancer, lung diseases, heart problems, stroke, psychiatric problems, and arthritis. Ascertainment therefore reflects physician-diagnosed conditions as reported by participants and standardised across waves by RAND.

**Table S1 Chronic condition list across datasets used in empirical analysis**

| **MIDUS** | **UKHLS** | **ELSA wave 4** | **HRS** |
| --- | --- | --- | --- |
| Heart disease | Asthma^*^ | High blood pressure or hypertension | High blood pressure |
| High blood pressure | Arthritis^*^ | Angina | Diabetes |
| Circulation problems | Congestive heart failure | Heart attack | Cancer^*^ |
| Blood clots | Coronary heart disease | Congestive heart failure | Lung diseases |
| Heart murmur | Angina | Heart murmur | Heart problems |
| TIA or stroke | Heart attack or myocardial infarction | Abnormal heart rhythm | Stroke |
| Anaemia or other blood disease | Stroke | Diabetes or high blood sugar | Psychiatric problems |
| Cholesterol problems | Emphysema^*^ | Stroke | Arthritis^*^ |
| Diabetes | Hyperthyroidism or an over-active thyroid^*^ | High cholesterol |  |
| Asthma^*^ | Hypothyroidism or an under-active thyroid^*^ | Chronic lung disease such as chronic bronchitis or emphysema^*^ |  |
| Emphysema/COPD^*^ | Chronic bronchitis^*^ | Asthma^*^ |  |
| Tuberculosis | Any kind of liver condition | Arthritis (including osteoarthritis, or rheumatism) ^*^ |  |
| Positive TB skin test | Cancer or malignancy^*^ | Osteoporosis, sometimes called thin or brittle bones |  |
| Thyroid disease^*^ | Diabetes | Cancer or a malignant tumour (excluding minor skin cancers) ^*^ |  |
| Peptic ulcer disease | Epilepsy | Parkinson's disease |  |
| Cancer^*^ | High blood pressure/hypertension | Any emotional, nervous or psychiatric problems |  |
| Colon polyp | Clinical depression | Alzheimer's disease |  |
| Arthritis^*^ |  | Dementia, organic brain syndrome, senility or any other serious memory impairment |  |
| Glaucoma |  |  |  |
| Cirrhosis/liver disease |  |  |  |
| Alcoholism |  |  |  |
| Depression |  |  |  |
| Blood transfusion before 1993 |  |  |  |

^*^Indicates diseases included in immune multimorbidity

**AL computation**

AL was constructed separately within each dataset using biomarkers commonly suggested for its measurement, with scoring approaches harmonised across studies.^6^ For all versions, biomarkers were coded as high-risk if they indicated dysregulation and 0 otherwise, and then summed to create participant-level scores (Table S1). Eight versions of allostatic load were generated: (1) simple count of high-risk biomarkers, (2) same as (1) but incorporating medication use so that biomarkers pharmacologically controlled were still coded as high-risk, (3) combining biomarkers with established clinical thresholds and those without, (4) same as (3) with medication incorporated, (5) a parsimonious score using CRP, resting heart rate, HDL-cholesterol, waist-to-height ratio, and HbA1c,^7^ (6) the parsimonious score with medication incorporated, (7) common biomarkers across all studies with risk quartiles determined by the pooled sample, and (8) the pooled sample score with medication incorporated.

**MIDUS**

In MIDUS, the allostatic load panel spanned multiple physiological systems: cardiovascular (systolic and diastolic blood pressure, resting pulse), metabolic (BMI, waist-to-height ratio, triglycerides, HDL-cholesterol, total cholesterol, HbA1c, fasting glucose, insulin resistance), kidney function (eGFR), inflammation and immune activity (CRP, IGF-1, IL-6, fibrinogen, intercellular and vascular adhesion molecules), neuroendocrine (urinary cortisol, urinary epinephrine and norepinephrine, DHEA-S), autonomic function (heart rate variability indices including SDRR, RMSSD, low- and high-frequency power), and respiratory function (peak expiratory flow). Medication data were linked to antihypertensives (blood pressure, pulse), glucose-lowering drugs (HbA1c, fasting glucose, insulin resistance), lipid-lowering drugs (HDL-cholesterol, triglycerides), anticoagulants/antiplatelets (fibrinogen), and respiratory medications (peak expiratory flow).

**UKHLS**

Biomarkers included hormonal (DHEA-S), cardiovascular (systolic and diastolic blood pressure, pulse), metabolic (BMI, waist-to-height ratio, triglycerides, HDL-cholesterol, HbA1c), kidney function (eGFR), inflammation (CRP, IGF-1, fibrinogen), and respiratory (peak expiratory flow). Medication information included antihypertensives (blood pressure, pulse), glucose-lowering therapies (HbA1c, fasting glucose), lipid-lowering drugs including statins (HDL-cholesterol, triglycerides), anticoagulants/antiplatelets (fibrinogen), and respiratory medications (peak expiratory flow)

**ELSA**

The panel included hormonal (DHEA-S), cardiovascular (systolic and diastolic blood pressure, pulse), metabolic (BMI, waist-to-height ratio, triglycerides, HDL-cholesterol, HbA1c, fasting glucose), inflammation (CRP, IGF-1, fibrinogen), and respiratory (peak expiratory flow). Medications considered were antihypertensives (blood pressure, pulse), glucose-lowering therapies (HbA1c, fasting glucose), lipid-lowering drugs (HDL-cholesterol, triglycerides), anticoagulants (fibrinogen), and respiratory medications (peak expiratory flow).

**HRS**

Available biomarkers included cardiovascular (systolic and diastolic blood pressure, pulse), metabolic (BMI, waist-to-height ratio, HDL-cholesterol, HbA1c), kidney function (cystatin C), inflammation (CRP), and respiratory (peak expiratory flow). Medication information included antihypertensives (blood pressure, pulse), glucose-lowering drugs (HbA1c), lipid-lowering drugs (HDL-cholesterol), and respiratory medications (peak expiratory flow).

**Pooled Sample**

In addition, a pooled allostatic load specification was constructed using biomarkers available across all datasets. Pooled allostatic load was based on SBP, DBP, pulse rate, BMI, waist-to-height ratio, HbA1c, HDL cholesterol, and CRP.

**Table S2 Biomarker thresholds and clinical cut-points for biomarkers used in the construction of allostatic load across data used in empirical analysis**

| **Dataset** | **MIDUS** | **UKHLS** | **ELSA** | **HRS** | **Clinical cutoffs** | **Respective diseases/use in FDA surrogate endpoint table** |
| --- | --- | --- | --- | --- | --- | --- |
| **AL** | | | | | | |
| **Cardiovascular** | | | | | | |
| SBP | ≥143 | ≥136.5 | ≥143.0 | ≥143.0 | ≥130 (US) / ≥140 (EU)^8,9^ | Hypertension^*^; Hypotension^*^; Interoperative haemorrhage |
| DBP | ≥82 | ≥80.0 | ≥81.5 | ≥86.5 | ≥80 (US) / ≥90 (EU)^8,9^ |  |
| Pulse rate | ≥77 | ≥75.0 | ≥66.5 | ≥77.000 | >80^10^ | Supraventricular tachycardia |
| **Metabolic - lipids** | | | | | | |
| BMI | ≥32.53 | ≥30.6 | ≥30.8 | ≥32.2 | ≥30^11^ |  |
| Waist to height ratio | ≥0.630 | ≥0.609 | ≥0.631 | ≥0.647 |  |  |
| Triglycerides | ≥156 (mg/dL) / ≥1.76 | ≥2.2 | ≥2.1 |  | ≥150 / ≥1.7^12^ | Familial chylomicronaemia syndrome; Severe hypertriglyceridemia; Lipodystrophy |
| HDL cholesterol | ≤43 (mg/dL) | ≤1.2 | ≤1.3 | ≤43.20 | <40 mg/dL /<1.0^13^ |  |
| **Metabolic - glucose metabolism** | | | | | | |
| Glycosylated haemoglobin (HbA1c) | ≥6.2 | ≥39 (mmol/mol) / ≥5.7 | ≥6.0 | ≥6.10 | ≥6.5% (≥48 mmol/mol)^14^ | Type 1 diabetes mellitus^*^; Type 2 diabetes mellitus^*^; Lipodystrophy |
| Fasting glucose | ≥104.5 (mg/dL) / ≥5.8 |  | ≥5.1 |  | >125 / >6.9^14^ | Lipodystrophy |
| Insulin resistance (HOMA-IR) | ≥4.28 |  |  |  |  |  |
| **Metabolic - other** | | | | | | |
| eGFR | ≤0.413 | ≤0.380 |  |  |  | Chronic kidney disease^*^; Active lupus nephritis |
| Cystatin C |  |  |  | ≥1.20 |  |  |
| **Inflammation** | | | | | | |
| CRP | ≥3.15 | ≥2.8 | ≥3.3 | ≥3.73 | >3^15^ |  |
| IGF-1 | ≤91 (ng/mL) / ≤11.9 | ≤14 | ≤12 |  |  | Acromegaly |
| IL6 | ≥3.272 |  |  |  |  |  |
| Fibrinogen | ≥392 (mg/dL) / ≥3.92 | ≥3.1 | ≥3.6 |  |  |  |
| sE-Selectin | ≥51.310 |  |  |  |  |  |
| sICAM-1 | ≥333.175 |  |  |  |  |  |
| **Sympathetic Nervous System** | | | | | | |
| Urine Epinephrine | ≥0.180 |  |  |  |  |  |
| Urine Norepinephrine | ≥2.50 |  |  |  |  |  |
| **Hypothalamic Pituitary Adrenal Axis** | | | | | | |
| Urine Cortisol | ≤0.429 |  |  |  |  | Cushing's disease; Endogenous Cushing’s syndrome |
| Blood DHEA-S | ≤52.0 (ng/mL) / ≤1.4 | ≤2.2 | ≤1.2 |  |  |  |
| **Parasympathetic Nervous System** | | | | | | |
| SDRR | ≤3.16 |  |  |  |  |  |
| RMSSD | ≤2.49 |  |  |  |  |  |
| Low frequency spectral power | ≤55.975 |  |  |  |  |  |
| High frequency spectral power | ≤4.028 |  |  |  |  |  |
| **Respiratory System** | | | | | | |
| Peak expiratory flow | ≤330 | ≤6 (per sec) / ≤360 | ≤279 | ≤270 |  |  |
| Note: Cells shaded in light orange denote instances in which the lowest quartile falls within the clinically defined high-risk range. | | | | | | |

^*^Diseases measured in at least one of the studies.

# Supplementary tables for results

**Table S3 System combination used in papers**

| **System combination** | **n** |
| --- | --- |
| Cardiovascular, HPA-Axis/Neuroendocrine, Immune/Inflammation, Metabolic | 105 |
| Cardiovascular, Immune/Inflammation, Metabolic | 86 |
| Cardiovascular, Immune/Inflammation, Liver Function, Metabolic, Renal/Kidney Function | 45 |
| Cardiovascular, Metabolic | 44 |
| Cardiovascular, HPA-Axis/Neuroendocrine, Metabolic | 39 |
| Cardiovascular, Immune/Inflammation, Liver Function, Metabolic | 29 |
| Cardiovascular, Immune/Inflammation, Metabolic, Renal/Kidney Function | 20 |
| Cardiovascular, HPA-Axis/Neuroendocrine, Immune/Inflammation, Metabolic, Renal/Kidney Function | 9 |
| Cardiovascular, HPA-Axis/Neuroendocrine, Immune/Inflammation, Liver Function, Metabolic | 8 |
| Cardiovascular, Liver Function, Metabolic, Renal/Kidney Function | 8 |
| Cardiovascular, HPA-Axis/Neuroendocrine, Immune/Inflammation, Liver Function, Metabolic, Renal/Kidney Function | 7 |
| Cardiovascular, Liver Function, Metabolic | 4 |
| Cardiovascular, Immune/Inflammation, Metabolic, Not Typically Allostatic Load Marker | 3 |
| HPA-Axis/Neuroendocrine | 3 |
| Immune/Inflammation | 3 |
| Cardiovascular, Immune/Inflammation, Liver Function, Renal/Kidney Function | 2 |
| Cardiovascular, Immune/Inflammation, Metabolic, Not Typically Allostatic Load Marker, Renal/Kidney Function | 2 |
| HPA-Axis/Neuroendocrine, Immune/Inflammation | 2 |
| Immune/Inflammation, Liver Function | 2 |
| Immune/Inflammation, Metabolic | 2 |
| Cardiovascular, HPA-Axis/Neuroendocrine | 1 |
| Cardiovascular, HPA-Axis/Neuroendocrine, Immune/Inflammation, Liver Function, Metabolic, Not Typically Allostatic Load Marker, Renal/Kidney Function | 1 |
| HPA-Axis/Neuroendocrine, Immune/Inflammation, Metabolic | 1 |
| HPA-Axis/Neuroendocrine, Immune/Inflammation, Renal/Kidney Function | 1 |
| HPA-Axis/Neuroendocrine, Metabolic | 1 |
| **Total** | **428** |

**Table S4 Biomarker combination used more than 5 times in papers^*^**

| **Biomarker combination** | **n** |
| --- | --- |
| BMI - Body Mass Index, Cortisol (All Measurement Types), DBP - Diastolic Blood Pressure, Epinephrine (Adrenaline), Norepinephrine (Noradrenaline), SBP - Systolic Blood Pressure | 15 |
| Cortisol (All Measurement Types), DBP - Diastolic Blood Pressure, DHEAS - Dehydroepiandrosterone, Epinephrine (Adrenaline), HbA1c - Glycated haemoglobin - Haemoglobin A1C, HDL-C - High Density Lipoprotein Cholesterol, Norepinephrine (Noradrenaline), SBP - Systolic Blood Pressure, TC - Total Cholesterol, Waist/Waist-to Hip Ratio/Waist-to-Height Ratio | 11 |
| Cortisol (All Measurement Types), CRP - C-Reactive Protein, DBP - Diastolic Blood Pressure, DHEAS - Dehydroepiandrosterone, Epinephrine (Adrenaline), HbA1c - Glycated haemoglobin - Haemoglobin A1C, HDL-C - High Density Lipoprotein Cholesterol, Norepinephrine (Noradrenaline), SBP - Systolic Blood Pressure, TC - Total Cholesterol, Waist/Waist-to Hip Ratio/Waist-to-Height Ratio | 10 |
| Albumin, CRP - C-Reactive Protein, DBP - Diastolic Blood Pressure, HbA1c - Glycated haemoglobin - Haemoglobin A1C, HDL-C - High Density Lipoprotein Cholesterol, RHR - Resting Heart Rate, SBP - Systolic Blood Pressure, TC - Total Cholesterol, Waist/Waist-to Hip Ratio/Waist-to-Height Ratio | 9 |
| BMI - Body Mass Index, Cortisol (All Measurement Types), CRP - C-Reactive Protein, DBP - Diastolic Blood Pressure, DHEAS - Dehydroepiandrosterone, E-Selectin, Epinephrine (Adrenaline), Fibrinogen - Clauss Fibrinogen, Glucose, HbA1c - Glycated haemoglobin - Haemoglobin A1C, HDL-C - High Density Lipoprotein Cholesterol, HF - High frequency spectral power, HOMA-IR - Homeostatic Model Assessment for Insulin Resistance, IL-6 - Interleukin-6, LDL-C - Low-Density Lipoprotein Cholesterol, LF - Low frequency spectral power, Norepinephrine (Noradrenaline), RHR - Resting Heart Rate, RMSSD - root mean square of successive differences between normal heartbeats, SBP - Systolic Blood Pressure, SDRR - Standard Deviation of R-R (heartbeat to heartbeat) intervals, Triglycerides, Waist/Waist-to Hip Ratio/Waist-to-Height Ratio | 8 |
| Albumin, BMI - Body Mass Index, CRP - C-Reactive Protein, DBP - Diastolic Blood Pressure, HbA1c - Glycated haemoglobin - Haemoglobin A1C, HDL-C - High Density Lipoprotein Cholesterol, RHR - Resting Heart Rate, SBP - Systolic Blood Pressure, TC - Total Cholesterol | 7 |
| Albumin, BMI - Body Mass Index, Creatinine Clearance, CRP - C-Reactive Protein, DBP - Diastolic Blood Pressure, HbA1c - Glycated haemoglobin - Haemoglobin A1C, HDL-C - High Density Lipoprotein Cholesterol, SBP - Systolic Blood Pressure, TC - Total Cholesterol, Triglycerides | 5 |
| BMI - Body Mass Index, CRP - C-Reactive Protein, DBP - Diastolic Blood Pressure, Glucose, HbA1c - Glycated haemoglobin - Haemoglobin A1C, HDL-C - High Density Lipoprotein Cholesterol, IL-6 - Interleukin-6, LDL-C - Low-Density Lipoprotein Cholesterol, Percent (%) body fat, SBP - Systolic Blood Pressure, TC - Total Cholesterol, TNF-α - Tumour-necrosis factor alpha, Triglycerides, Waist/Waist-to Hip Ratio/Waist-to-Height Ratio | 5 |
| BMI - Body Mass Index, Cortisol (All Measurement Types), CRP - C-Reactive Protein, DHEAS - Dehydroepiandrosterone, E-Selectin, Epinephrine (Adrenaline), Fibrinogen - Clauss Fibrinogen, Glucose, HbA1c - Glycated haemoglobin - Haemoglobin A1C, HDL-C - High Density Lipoprotein Cholesterol, HF - High frequency spectral power, HOMA-IR - Homeostatic Model Assessment for Insulin Resistance, IL-6 - Interleukin-6, LDL-C - Low-Density Lipoprotein Cholesterol, LF - Low frequency spectral power, Norepinephrine (Noradrenaline), PP - Pulse Pressure, RHR - Resting Heart Rate, RMSSD - root mean square of successive differences between normal heartbeats, SBP - Systolic Blood Pressure, SDRR - Standard Deviation of R-R (heartbeat to heartbeat) intervals, Triglycerides, Waist/Waist-to Hip Ratio/Waist-to-Height Ratio | 5 |
| Creatinine Clearance, CRP - C-Reactive Protein, DBP - Diastolic Blood Pressure, DHEAS - Dehydroepiandrosterone, Fibrinogen - Clauss Fibrinogen, HbA1c - Glycated haemoglobin - Haemoglobin A1C, IGF1 - Insulin-Like Growth Factor 1, RHR - Resting Heart Rate, SBP - Systolic Blood Pressure, TC/HDL - Total cholesterol to High Density Lipoprotein cholesterol ratio, Triglycerides, Waist/Waist-to Hip Ratio/Waist-to-Height Ratio | 5 |
| DBP - Diastolic Blood Pressure, Glucose, HDL-C - High Density Lipoprotein Cholesterol, SBP - Systolic Blood Pressure, Triglycerides, Waist/Waist-to Hip Ratio/Waist-to-Height Ratio | 5 |

^*^Biomarker combinations used less than 5 times were not listed.

**Table S5 Heterogeneity in the operationalisation of allostatic load**

| **AL Method Group Name** | **Count (Papers that used Method Group in any of their datasets)** |
| --- | --- |
| Count Risk Score | 311 |
| Standardised Score | 56 |
| Systems Risk Score | 42 |
| Count Risk Score - converted to dummy variable | 28 |
| Measurement and Path Models | 21 |
| Risk Score per System | 13 |
| Biomarkers Analysed Separately | 11 |
| Case Based Profiles | 5 |
| Average Risk Score | 2 |
| Other Method Details | 1 |

**Table S6 C-statistics and ΔC-statistics (95% CIs) for alternative specifications of allostatic load in relation to general and cardiometabolic multimorbidity across datasets**

| **Dataset** | **Outcome** | **Model** | **C-statistic** | **ΔC-statistic (95% CI)** |
| --- | --- | --- | --- | --- |
| ELSA | General multimorbidity | Allostatic load (no med-adjusted, reference) | 0.69 (0.67–0.72) | ref |
|  |  | Allostatic load (med-adjusted) | 0.79 (0.77–0.81) | 0.09 (0.08, 0.11) |
|  |  | Allostatic load (clinical cutoffs, no med-adjusted) | 0.69 (0.67–0.72) | -0.00 (-0.00, 0.00) |
|  |  | Allostatic load (clinical cutoffs, med-adjusted) | 0.78 (0.76–0.80) | 0.08 (0.07, 0.10) |
|  |  | Five-item allostatic load (no med-adjusted) | 0.68 (0.66–0.70) | -0.01 (-0.02, -0.00) |
|  |  | Five-item allostatic load (med-adjusted) | 0.76 (0.74–0.78) | 0.06 (0.05, 0.08) |
|  |  | Pooled common-biomarker AL | 0.68 (0.66, 0.71) | -0.01 (-0.02, -0.00) |
|  |  | Pooled common-biomarker AL (med-adjusted) | 0.78 (0.76, 0.80) | 0.09 (0.07, 0.10) |
|  | Cardiometabolic multimorbidity | Allostatic load (no med-adjusted, reference) | 0.70 (0.68–0.73) | ref |
|  |  | Allostatic load (med-adjusted) | 0.85 (0.84–0.87) | 0.15 (0.13, 0.17) |
|  |  | Allostatic load (clinical cutoffs, no med-adjusted) | 0.71 (0.68–0.73) | 0.00 (-0.00, 0.01) |
|  |  | Allostatic load (clinical cutoffs, med-adjusted) | 0.84 (0.82–0.86) | 0.14 (0.12, 0.16) |
|  |  | Five-item allostatic load (no med-adjusted) | 0.70 (0.67–0.72) | -0.01 (-0.02, 0.00) |
|  |  | Five-item allostatic load (med-adjusted) | 0.81 (0.79–0.83) | 0.11 (0.09, 0.13) |
|  |  | Pooled common-biomarker AL | 0.69 (0.67, 0.72) | -0.01 (-0.02, 0.00) |
|  |  | Pooled common-biomarker AL (med-adjusted) | 0.85 (0.83, 0.87) | 0.15 (0.13, 0.17) |
|  | Immune multimorbidity | Allostatic load (no med-adjusted, reference) | 0.69 (0.64–0.74) | ref |
|  |  | Allostatic load (med-adjusted) | 0.70 (0.65–0.74) | 0.01 (-0.00, 0.03) |
|  |  | Allostatic load (clinical cutoffs, no med-adjusted) | 0.69 (0.64–0.74) | 0.00 (-0.00, 0.01) |
|  |  | Allostatic load (clinical cutoffs, med-adjusted) | 0.70 (0.66–0.75) | 0.01 (-0.00, 0.03) |
|  |  | Five-item allostatic load (no med-adjusted) | 0.69 (0.65–0.74) | 0.00 (-0.01, 0.02) |
|  |  | Five-item allostatic load (med-adjusted) | 0.70 (0.66–0.75) | 0.01 (-0.00, 0.03) |
|  |  | Pooled common-biomarker AL (no med-adjusted) | 0.68 (0.64–0.73) | -0.00 (-0.02, 0.01) |
|  |  | Pooled common-biomarker AL (med-adjusted) | 0.69 (0.64–0.74) | 0.00 (-0.02, 0.02) |
| HRS | General multimorbidity | Allostatic load (no med-adjusted, reference) | 0.72 (0.70–0.73) | ref |
|  |  | Allostatic load (med-adjusted) | 0.82 (0.81–0.83) | 0.10 (0.09, 0.11) |
|  |  | Allostatic load (clinical cutoffs, no med-adjusted) | 0.71 (0.70–0.72) | -0.01 (-0.01, -0.00) |
|  |  | Allostatic load (clinical cutoffs, med-adjusted) | 0.80 (0.79–0.81) | 0.09 (0.08, 0.10) |
|  |  | Five-item allostatic load (no med-adjusted) | 0.70 (0.69–0.71) | -0.01 (-0.02, -0.01) |
|  |  | Five-item allostatic load (med-adjusted) | 0.78 (0.77–0.79) | 0.07 (0.06, 0.07) |
|  |  | Pooled common-biomarker AL | 0.70 (0.69, 0.72) | -0.01 (-0.02, -0.01) |
|  |  | Pooled common-biomarker AL (med-adjusted) | 0.80 (0.79, 0.81) | 0.09 (0.08, 0.09) |
|  | Cardiometabolic multimorbidity | Allostatic load (no med-adjusted, reference) | 0.72 (0.70–0.73) | ref |
|  |  | Allostatic load (med-adjusted) | 0.84 (0.83–0.85) | 0.12 (0.11, 0.13) |
|  |  | Allostatic load (clinical cutoffs, no med-adjusted) | 0.71 (0.70–0.72) | -0.01 (-0.01, -0.00) |
|  |  | Allostatic load (clinical cutoffs, med-adjusted) | 0.82 (0.81–0.83) | 0.11 (0.10, 0.12) |
|  |  | Five-item allostatic load (no med-adjusted) | 0.71 (0.70–0.72) | -0.01 (-0.01, -0.00) |
|  |  | Five-item allostatic load (med-adjusted) | 0.82 (0.81–0.83) | 0.10 (0.09, 0.11) |
|  |  | Pooled common-biomarker AL | 0.70 (0.69, 0.71) | -0.02 (-0.02, -0.01) |
|  |  | Pooled common-biomarker AL (med-adjusted) | 0.82 (0.81, 0.83) | 0.10 (0.10, 0.11) |
|  | Immune multimorbidity | Allostatic load (no med-adjusted, reference) | 0.65 (0.64–0.67) | ref |
|  |  | Allostatic load (med-adjusted) | 0.66 (0.64–0.67) | 0.01 (-0.00, 0.01) |
|  |  | Allostatic load (clinical cutoffs, no med-adjusted) | 0.65 (0.63–0.66) | -0.00 (-0.01, -0.00) |
|  |  | Allostatic load (clinical cutoffs, med-adjusted) | 0.65 (0.63–0.66) | -0.00 (-0.01, 0.00) |
|  |  | Five-item allostatic load (no med-adjusted) | 0.64 (0.63–0.66) | -0.01 (-0.01, -0.00) |
|  |  | Five-item allostatic load (med-adjusted) | 0.65 (0.63–0.66) | -0.01 (-0.01, 0.00) |
|  |  | Pooled common-biomarker AL (no med-adjusted) | 0.64 (0.63–0.66) | -0.01 (-0.01, -0.00) |
|  |  | Pooled common-biomarker AL (med-adjusted) | 0.64 (0.63–0.66) | -0.01 (-0.01, -0.00) |
| MIDUS | General multimorbidity | Allostatic load (no med-adjusted, reference) | 0.76 (0.72–0.80) | ref |
|  |  | Allostatic load (med-adjusted) | 0.79 (0.76–0.83) | 0.03 (0.02, 0.05) |
|  |  | Allostatic load (clinical cutoffs, no med-adjusted) | 0.76 (0.72–0.80) | 0.01 (0.00, 0.01) |
|  |  | Allostatic load (clinical cutoffs, med-adjusted) | 0.79 (0.76–0.83) | 0.03 (0.02, 0.05) |
|  |  | Five-item allostatic load (no med-adjusted) | 0.74 (0.70–0.78) | -0.02 (-0.03, 0.00) |
|  |  | Five-item allostatic load (med-adjusted) | 0.78 (0.74–0.82) | 0.02 (-0.00, 0.04) |
|  |  | Pooled common-biomarker AL | 0.74 (0.70, 0.78) | -0.02 (-0.04, 0.00) |
|  |  | Pooled common-biomarker AL (med-adjusted) | 0.78 (0.74, 0.82) | 0.02 (0.00, 0.04) |
|  | Cardiometabolic multimorbidity | Allostatic load (no med-adjusted, reference) | 0.75 (0.72–0.78) | ref |
|  |  | Allostatic load (med-adjusted) | 0.83 (0.80–0.86) | 0.08 (0.06, 0.10) |
|  |  | Allostatic load (clinical cutoffs, no med-adjusted) | 0.75 (0.72–0.78) | 0.00 (-0.00, 0.01) |
|  |  | Allostatic load (clinical cutoffs, med-adjusted) | 0.82 (0.80–0.85) | 0.08 (0.06, 0.09) |
|  |  | Five-item allostatic load (no med-adjusted) | 0.75 (0.72–0.78) | -0.00 (-0.02, 0.02) |
|  |  | Five-item allostatic load (med-adjusted) | 0.84 (0.81–0.87) | 0.09 (0.07, 0.11) |
|  |  | Pooled common-biomarker AL | 0.74 (0.71, 0.78) | -0.01 (-0.03, 0.01) |
|  |  | Pooled common-biomarker AL (med-adjusted) | 0.85 (0.82, 0.87) | 0.10 (0.07, 0.12) |
|  | Immune multimorbidity | Allostatic load (no med-adjusted, reference) | 0.70 (0.66–0.75) | ref |
|  |  | Allostatic load (med-adjusted) | 0.71 (0.66–0.75) | 0.01 (-0.00, 0.01) |
|  |  | Allostatic load (clinical cutoffs, no med-adjusted) | 0.70 (0.66–0.74) | -0.00 (-0.00, 0.00) |
|  |  | Allostatic load (clinical cutoffs, med-adjusted) | 0.71 (0.66–0.75) | 0.00 (-0.00, 0.01) |
|  |  | Five-item allostatic load (no med-adjusted) | 0.70 (0.66–0.75) | -0.00 (-0.01, 0.01) |
|  |  | Five-item allostatic load (med-adjusted) | 0.71 (0.66–0.75) | 0.01 (-0.01, 0.02) |
|  |  | Pooled common-biomarker AL (no med-adjusted) | 0.70 (0.66–0.74) | -0.00 (-0.01, 0.01) |
|  |  | Pooled common-biomarker AL (med-adjusted) | 0.71 (0.66–0.75) | 0.01 (-0.01, 0.02) |
| UKHLS | General multimorbidity | Allostatic load (no med-adjusted, reference) | 0.78 (0.76–0.79) | ref |
|  |  | Allostatic load (med-adjusted) | 0.80 (0.79–0.82) | 0.02 (0.02, 0.03) |
|  |  | Allostatic load (clinical cutoffs, no med-adjusted) | 0.78 (0.77–0.80) | 0.00 (0.00, 0.00) |
|  |  | Allostatic load (clinical cutoffs, med-adjusted) | 0.80 (0.79–0.81) | 0.02 (0.02, 0.03) |
|  |  | Five-item allostatic load (no med-adjusted) | 0.78 (0.76–0.79) | -0.00 (-0.01, 0.00) |
|  |  | Five-item allostatic load (med-adjusted) | 0.80 (0.78–0.81) | 0.02 (0.01, 0.03) |
|  |  | Pooled common-biomarker AL | 0.77 (0.76, 0.79) | -0.00 (-0.01, 0.00) |
|  |  | Pooled common-biomarker AL (med-adjusted) | 0.80 (0.78, 0.81) | 0.02 (0.01, 0.02) |
|  | Cardiometabolic multimorbidity | Allostatic load (no med-adjusted, reference) | 0.82 (0.80–0.84) | ref |
|  |  | Allostatic load (med-adjusted) | 0.85 (0.83–0.87) | 0.03 (0.02, 0.03) |
|  |  | Allostatic load (clinical cutoffs, no med-adjusted) | 0.82 (0.80–0.84) | 0.00 (-0.00, 0.00) |
|  |  | Allostatic load (clinical cutoffs, med-adjusted) | 0.85 (0.83–0.87) | 0.03 (0.02, 0.04) |
|  |  | Five-item allostatic load (no med-adjusted) | 0.84 (0.82–0.86) | 0.02 (0.01, 0.03) |
|  |  | Five-item allostatic load (med-adjusted) | 0.86 (0.84–0.88) | 0.04 (0.03, 0.05) |
|  |  | Pooled common-biomarker AL | 0.83 (0.81, 0.85) | 0.01 (0.00, 0.02) |
|  |  | Pooled common-biomarker AL (med-adjusted) | 0.85 (0.83, 0.87) | 0.03 (0.02, 0.04) |
|  | Immune multimorbidity | Allostatic load (no med-adjusted, reference) | 0.75 (0.72–0.77) | ref |
|  |  | Allostatic load (med-adjusted) | 0.77 (0.74–0.79) | 0.02 (0.01, 0.03) |
|  |  | Allostatic load (clinical cutoffs, no med-adjusted) | 0.74 (0.72–0.77) | -0.00 (-0.00, 0.00) |
|  |  | Allostatic load (clinical cutoffs, med-adjusted) | 0.76 (0.73–0.78) | 0.01 (0.01, 0.02) |
|  |  | Five-item allostatic load (no med-adjusted) | 0.73 (0.71–0.76) | -0.01 (-0.02, -0.00) |
|  |  | Five-item allostatic load (med-adjusted) | 0.75 (0.73–0.78) | 0.01 (-0.00, 0.02) |
|  |  | Pooled common-biomarker AL (no med-adjusted) | 0.74 (0.71–0.76) | -0.01 (-0.02, -0.00) |
|  |  | Pooled common-biomarker AL (med-adjusted) | 0.76 (0.73–0.78) | 0.01 (0.00, 0.02) |

# Reference

1.	University of Essex I for S. United Kingdom Household Longitudinal Study; UKHLSUnderstanding Society: Waves 1- , 2008-Understanding Society: Waves 2-3 Nurse Health Assessment, 2010-2012. Published online 2025. doi:10.5255/UKDA-SN-7251-6

2.	Banks J, Batty GD, Breedvelt J, et al. ELSAEnglish Longitudinal Study of AgeingEnglish Longitudinal Study of Ageing: Waves 0-11, 1998-2024. Published online 2025. doi:10.5255/UKDA-SN-5050-33

3.	Sonnega A, Faul JD, Ofstedal MB, Langa KM, Phillips JW, Weir DR. Cohort Profile: the Health and Retirement Study (HRS). *Int J Epidemiol*. 2014;43(2):576-585. doi:10.1093/ije/dyu067

4.	Ryff CD, Seeman T, Weinstein M. Midlife in the United States (MIDUS 2): Biomarker Project, 2004-2009. Published online 2025. doi:10.3886/ICPSR29282.v11

5.	Bugliari D, Carroll J, Hayden O, et al. *RAND HRS Longitudinal File 2020 (V2) Documentation: Includes 1992-2020 (Final Release)*.; 2024. Accessed August 21, 2025. https://www.rand.org/pubs/tools/TLA2097-1-v3.html

6.	Juster RP, McEwen BS, Lupien SJ. Allostatic load biomarkers of chronic stress and impact on health and cognition. *Neuroscience & Biobehavioral Reviews*. 2010;35(1):2-16. doi:10.1016/j.neubiorev.2009.10.002

7.	McCrory C, McLoughlin S, Layte R, et al. Towards a consensus definition of allostatic load: a multi-cohort, multi-system, multi-biomarker individual participant data (IPD) meta-analysis. *Psychoneuroendocrinology*. 2023;153:106117. doi:10.1016/j.psyneuen.2023.106117

8.	Whelton PK, Carey RM, Aronow WS, et al. 2017 ACC/AHA/AAPA/ABC/ACPM/AGS/APhA/ASH/ASPC/NMA/PCNA Guideline for the Prevention, Detection, Evaluation, and Management of High Blood Pressure in Adults: Executive Summary: A Report of the American College of Cardiology/American Heart Association Task Force on Clinical Practice Guidelines. *Hypertension*. 2018;71(6):1269-1324. doi:10.1161/HYP.0000000000000066

9.	Mancia G, Kreutz R, Brunström M, et al. 2023 ESH Guidelines for the management of arterial hypertension The Task Force for the management of arterial hypertension of the European Society of Hypertension: Endorsed by the International Society of Hypertension (ISH) and the European Renal Association (ERA). *J Hypertens*. 2023;41(12):1874-2071. doi:10.1097/HJH.0000000000003480

10.	Williams B, Mancia G, Spiering W, et al. 2018 ESC/ESH Guidelines for the management of arterial hypertension: The Task Force for the management of arterial hypertension of the European Society of Cardiology (ESC) and the European Society of Hypertension (ESH). *Eur Heart J*. 2018;39(33):3021-3104. doi:10.1093/eurheartj/ehy339

11.	Potter AW, Chin GC, Looney DP, Friedl KE. Defining Overweight and Obesity by Percent Body Fat Instead of Body Mass Index. *J Clin Endocrinol Metab*. 2024;110(4):e1103-e1107. doi:10.1210/clinem/dgae341

12.	G. Parhofer K, Laufs U. The Diagnosis and Treatment of Hypertriglyceridemia. *Dtsch Arztebl Int*. 2019;116(49):825-832. doi:10.3238/arztebl.2019.0825

13.	Expert Panel on Detection E and Treatment of High Blood Cholesterol in Adults. Executive Summary of the Third Report of the National Cholesterol Education Program (NCEP) Expert Panel on Detection, Evaluation, and Treatment of High Blood Cholesterol in Adults (Adult Treatment Panel III). *JAMA*. 2001;285(19):2486-2497. doi:10.1001/jama.285.19.2486

14.	American Diabetes Association Professional Practice Committee. 2. Diagnosis and Classification of Diabetes: Standards of Care in Diabetes—2025. *Diabetes Care*. 2024;48(Supplement_1):S27-S49. doi:10.2337/dc25-S002

15.	Singh B, Goyal A, Patel BC. C-Reactive Protein: Clinical Relevance and Interpretation. In: *StatPearls*. StatPearls Publishing; 2025. Accessed August 21, 2025. http://www.ncbi.nlm.nih.gov/books/NBK441843/
